# Does smoking affect the outcomes of lumbar decompression surgery?

**DOI:** 10.1051/sicotj/2017050

**Published:** 2017-11-22

**Authors:** Radha Mehta, Himanshu Sharma

**Affiliations:** 1 Plymouth University Penisula School of Medicine and Dentistry, Tamar Science Park, Research Way Plymouth Devon PL6 8DH UK; 2 Southwest Neurosurgery Centre, Derriford Hospital Plymouth PL6 8DH UK

**Keywords:** Micro-discectomy, Spinal decompression, Smoking, Non-complex spinal surgery

## Abstract

*Introduction*: Lumbar decompressions and micro-discectomies are commonly performed non-complex spinal surgeries that do not involve the insertion of metalwork into the spine and are done for symptomatic disc prolapse and lumbar spinal stenosis, whereas complex-spinal surgery does require metalwork [1]. Studies of complex-spinal surgeries show that smoking has a significant negative impact on the outcome of the surgery [2] therefore, the cessation of smoking is advised prior to surgery [3]. There are evidences in the literature supportive as well as opposing this statement about continued smoking and poor outcome of decompressive spinal surgeries.

*Methods*: We retrospectively reviewed 143 consecutive patients who have had either a micro-discectomy or a micro-decompression.

*Results*: We found no statistical difference between smokers and non-smokers in the outcomes of lumbar decompression surgery. Both groups improved equally and significantly in terms of back pain, leg pain and functions. Out of 143 patients, only 2% more non-smokers had improved leg pain compared to smokers, 1% less non-smokers had improved back pain and 2% more non-smokers had an improved Oswestry Disability Index (ODI) score.

*Discussion*: We recommend that it is important to surgically treat both smokers and non-smokers in need of a lumbar spinal decompression.

## Introduction

Lumbar disc herniation and spinal stenosis can cause debilitating nerve pain, paraesthesia and muscle weakness. Such problems may lead to chronic pain and immobility, which in turn can cause difficulties in work, exercise and personal life [4]. It is therefore important to treat the problem, by performing micro-discectomies and spinal decompressions in patients with persistent and/or progressive sciatica.

There are a number of health risks and co-morbidities associated with smoking which could be associated with poor outcome in complex-spinal surgery. It is vital to investigate whether there are similar problems associated with smoking in lumbar spinal decompressions. This study aimed to evaluate whether smoking has any effect on the outcome of spinal decompressions or micro-discectomies. The study evaluated two groups, smokers and non-smokers who have had a lumbar decompression for disc prolapse and spinal stenosis and compared their leg pain, back pain, Oswestry Disability Index (ODI) pre- and post-operatively. We also analysed whether there were any peri-operative complications following spinal surgery in smokers compared to non-smokers.

## Materials and methods

### Patients

This is a retrospective study using a database of 143 consecutive patients who have had either a micro-discectomy or a decompression between August 2012 and September 2014. From the 143 patients used in the data, 70 were male and 73 female with the median age being 58 years. There were 67 patients who had a micro-discectomy and 76 had a decompression procedure, with a mix of single-level and multi-level disease. All surgeries were carried out by the same surgeon.

The patients were therefore split up into two groups; Smokers and Non-Smokers (including ex-smokers). The term ex-smoker refers to an individual who has given up cigarette and tobacco smoking, whereas a current smoker is an individual who has smoked at least 100 cigarettes in their lifetime and is a current smoker [5].

Sixty-six patients were smokers or had a history of smoking, and 77 were non-smokers and had no history of smoking. The smoker patients’ Pack Years ranged from one to 50 with an average of 25 pack years; the median number of cigarettes smoked per day was 20. There were only six patients that smoked less than five cigarettes a day.

### Treatment

All operations were carried out by the same surgeon. A standard micro-discectomy approach was used with midline incision, ipsilateral paraspinal dissection, minimal exposure laminotomy, and microscope-assisted discectomy and/or lateral recess decompression with undercutting facetectomy [6].

### Data collection

The data was collected using patient notes, clinical letters, operation notes and discharge letters. The information collected included age, sex, date of birth, type of operation (micro-discectomy or a decompression) and smoking history (whether or not they are smokers, and if so how many cigarettes they smoke a day and for how long have they smoked). If they were ex-smokers, then data was based on when did they quit, how many did they previously smoke per day and for how long. Pre-operative leg pain using a Visual Analogue Scale (VAS-LP) and back pain measured using a Visual Analogue Scale (VAS-BP) were recorded. From a scale of 0–10, these included; “*Pain when worst being 10*”, “*pain when least being 0*”, and “*pain right now*”. The mean value of the three scales was recorded as VAS leg pain and VAS back pain [7]. Functional disability caused by the pain was measured using ODI (Oswestry Disability Index) [8]. This was measured using a Questionnaire [9, 10]. This questionnaire asks ten questions, which each have a score of 1–5, to indicate how debilitating the functions are. The questions asked investigated how the pain affects their sleeping, their personal care, their ability to walk, etc. The answer was divided by 50 and multiplied by 100 to give an overall percentage. Post-operative data were then collected on any complications peri-operatively, on post-operative lower back pain using VAS-BP [7], post-op leg pain using VAS-LP [7] and post-op disability caused by pain, measured using an Oswestry Lower Back Pain Disability Questionnaire [9]. An improvement equal to or more than 20% in the ODI score and two or more out of ten in the VAS was considered clinically significant. We also measured the duration of follow-up for each of these patient groups.

### Statistical analysis

The data was analysed using a *χ* ^2^ test on SPSS software. The *p*-value of < 0.05 was considered as statistically significant.

## Results

Overall, our results (Table 1) showed 2% more non-smokers had improved leg pain compared to smokers, 1% more non-smokers had improved back pain and 6% less non-smokers had an improved ODI compared to smokers. Only one patient with a history of smoking experienced post-operative complication (2%), which was a chest complication secondary to smoking, and none of the non-smoker patients experienced any peri-operative complications.

**Table 1.** Shows the percentage of smokers and non-smokers that improved VAS-LP, VAS-BP, ODI and peri-operative complication.

	Smokers (%)	Non-smokers (%)
Percentage of patients with improved VAS-LP	86	88
Percentage of patients with improved VAS-BP	79	78
Percentage of patients with improved ODI	88	82
Percentage of patients that experienced peri-operative complication	2	0

In our study, *p*-value for the outcome of leg pain was 0.6, for back pain as 1.0 and ODI as 0.7. In all cases, the *p*-value was > 0.05 and the null hypothesis remained true as no statistical difference between smokers and non-smokers in the outcomes of non-complex decompressive spinal surgery.

**Figure 1.**
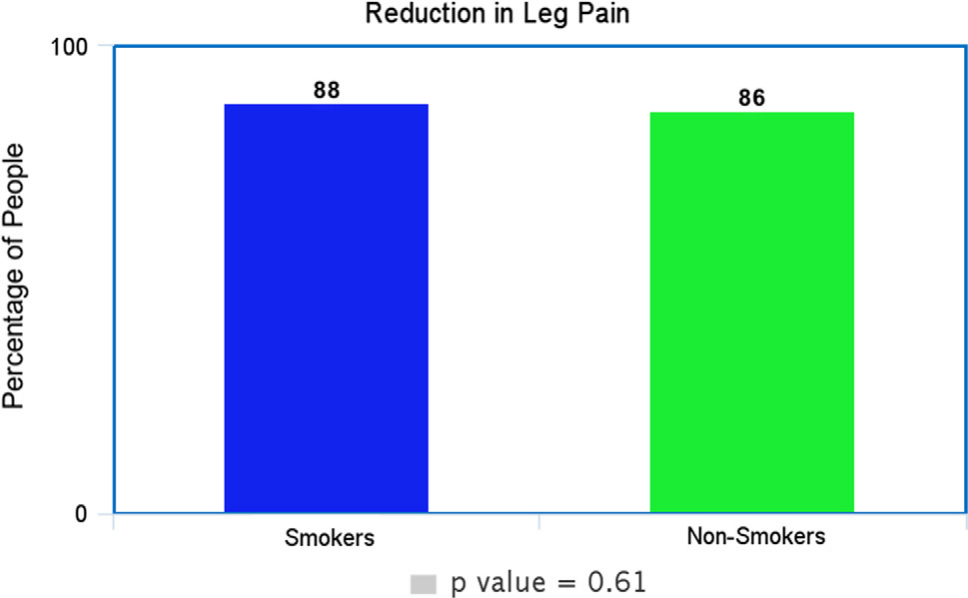
Shows the percentage of smokers and non-smokers that decreased at least two points in the VAS-LP.

**Figure 2.**
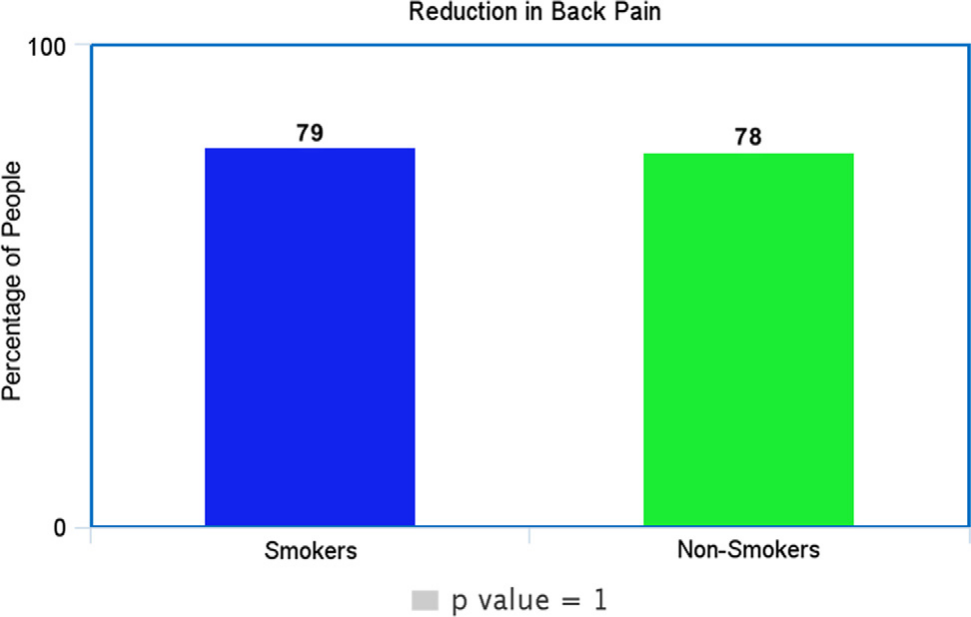
Shows the percentage of smokers and non-smokers that decreased at least two points Back Pain.

**Figure 3.**
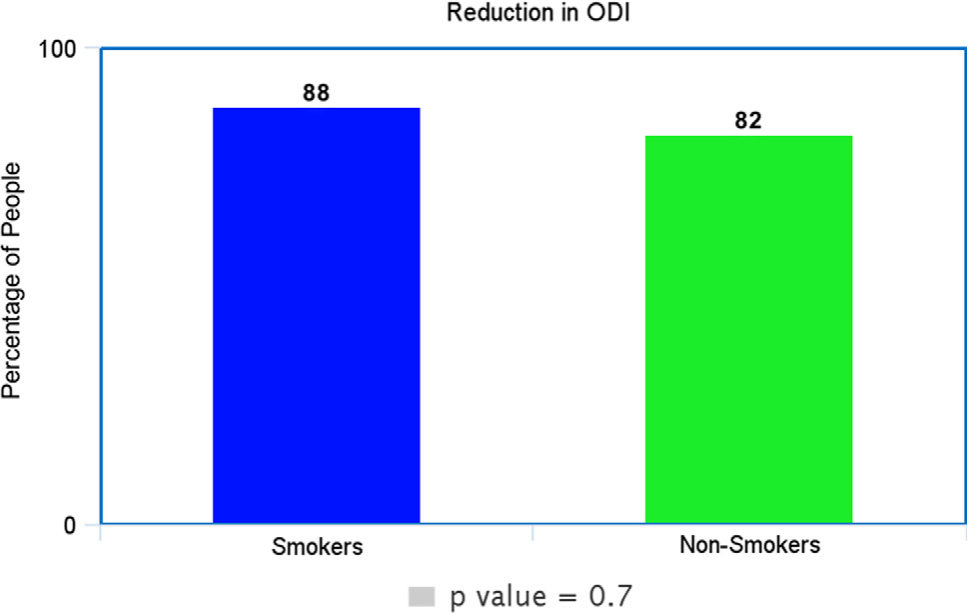
Shows the percentage of smokers and non-smokers that decreased at least 20% in the ODI questionnaire.

Each of these Figures 1–3 shows comparable clinical results with very little difference in the clinical and subjective outcome between smokers and non-smokers.

This was further evidenced by the mean follow-up duration between smokers and non-smokers; 6.7 months for the smoking group and 6.4 months for the non-smoking group, showing a non-significant difference.

## Discussion

This study demonstrated that there was no difference in the clinical as well as subjective outcome of lumbar decompressions and micro-discectomies for smokers and non-smokers.

There are evidences in the literature supportive as well as opposing this statement. In a retrospective cohort study by Appaduray and Lo [11] looking at the effects of diabetes and smoking as variables on the outcome of lumbar surgery. Of 902 patients who underwent lumbar spinal surgery between 2001 and 2005, inclusively, this paper concluded that diabetes does increase the risk of poor outcome following lumbar spinal surgery. However, no association was found between a positive smoking history and an increased incidence of poor outcome. Gulati et al. published a study questioning whether daily smoking affects outcomes after micro-decompression for degenerative central lumbar spinal stenosis (LSS) [12]. A total of 825 patients were included (619 non-smokers and 206 smokers). It concluded that non-smokers experienced a significantly greater improvement at one year following micro-decompression for LSS compared to smokers. However, it was noted that a considerable improvement was also found amongst smokers. This shows that smoking may not necessarily have been the cause for deterioration.

However, Nerland et al. published a paper showing opposing results. This study looked at different predictors of deterioration after decompressive surgery for lumbar spinal stenosis [13]. It used 1735 patients with complete 12 months follow-up and found that both old age and smoking together caused deterioration of pain and disability, shown by a decreasing post-operative ODI. However, by studying old age and smoking together as variables, it does not show whether smoking was an independent predictor for deterioration, or whether this was due to old age.

Ideally in a future version of this study, we would subdivide the smokers group further into heavy and light smokers, to see if we could demonstrate a risk of complications at higher smoking exposure. However, this information was not available to us in this retrospective study, and patient numbers per group may not have been large enough for us to establish significant results.

Nevertheless, smoking could be associated with increased risk of chest complications, venous thrombo-embolism and wound complications. Noteworthy is that decompressive lumbar spinal procedures are minor insult to body’s metabolic and physiological responses. They could be treated as a day case or overnight admission in vast majority of cases. This study revealed that there was no significant difference in the outcome of lumbar decompressions between smokers and non-smokers with regard to improvement in pain and functions. We also confirmed that medical and surgical complications were also comparable and negligible in the two studied groups. Amount and duration of smoking status did not show any difference in the outcome. By unnecessarily refusing treatment, patients may encounter chronic, debilitating nerve pain, which can cause further problems such as job loss, depression and further immobility [14]. Nevertheless, due to the co-morbidities associated with smoking, smoking should be discouraged in patients [15]. We conclude that lumbar spinal decompressions and micro-discectomies could be offered to patients with persistent and/or progressive sciatica regardless of their smoking status.

## Conflict of interest

The authors declare that they have no conflict of interest in relation with this paper.
